# Combining Costs and Benefits of Animal Activities to Assess Net Yield Outcomes in Apple Orchards

**DOI:** 10.1371/journal.pone.0158618

**Published:** 2016-07-08

**Authors:** Manu E. Saunders, Gary W. Luck

**Affiliations:** Institute for Land Water and Society, Charles Sturt University, PO Box 789, Albury, NSW, 2640, Australia; University of Northampton, UNITED KINGDOM

## Abstract

Diverse animal communities influence ecosystem function in agroecosystems through positive and negative plant-animal interactions. Yet, past research has largely failed to examine multiple interactions that can have opposing impacts on agricultural production in a given context. We collected data on arthropod communities and yield quality and quantity parameters (fruit set, yield loss and net outcomes) in three major apple-growing regions in south-eastern Australia. We quantified the net yield outcome (accounting for positive and negative interactions) of multiple animal activities (pollination, fruit damage, biological control) across the entire growing season on netted branches, which excluded vertebrate predators of arthropods, and open branches. Net outcome was calculated as the number of undamaged fruit at harvest as a proportion of the number of blossoms (i.e., potential fruit yield). Vertebrate exclusion resulted in lower levels of fruit set and higher levels of arthropod damage to apples, but did not affect net outcomes. Yield quality and quantity parameters (fruit set, yield loss, net outcomes) were not directly associated with arthropod functional groups. Model variance and significant differences between the ratio of pest to beneficial arthropods between regions indicated that complex relationships between environmental factors and multiple animal interactions have a combined effect on yield. Our results show that focusing on a single crop stage, species group or ecosystem function/service can overlook important complexity in ecological processes within the system. Accounting for this complexity and quantifying the net outcome of ecological interactions within the system, is more informative for research and management of biodiversity and ecosystem services in agricultural landscapes.

## Introduction

Sustainable management of agroecosystems requires a clear understanding of the ecology of species interactions and how they influence ecosystem services within the system. Provision of ecosystem services depends on complex interactions between species ecology, environmental factors, farm management and animal community dynamics [1, 2]. Traditionally, each ecosystem service and its associated biotic community have been studied separately (e.g. pollination services and pollinator insect communities; or pest control services and pest/predator communities) [3]. Yet, because the outcome of individual interactions can change relative to the outcomes of other interactions, as well as under different environmental contexts, studying ecosystem services individually can overlook important ecological complexity. A more informative approach is to assess net outcomes across space and time, whereby trade-offs in the activities of different species are accounted for, including both ecosystem services and disservices [4]. This approach is similar to modelling ‘bundles’ of services, and the trade-offs between them, spatially across a landscape, e.g. [5], but our emphasis here is on the ecological dynamics occurring within a single ecosystem.

In agroecosystems, pollination and pest control can complement each other in an additive or synergistic way [2, 6]. Pollinators enhance fruit set, directly benefitting final yields, while predators and parasitoids can control damaging insect pests, thereby indirectly contributing to final yields. However, the ecological interactions that lead to pollination and pest control services do not occur in isolation. Multiple plant and animal species interact within a crop system in complex ways, and the consequences of this for crop production depend on the species involved, the magnitude of their effects on different stages of the crop cycle, and the influence of management factors. For example, fruit set may decline as a result of flower damage by insect pests, regardless of pollinator abundance or effectiveness [7]. After fruit set, the benefits gained from pollination services during flowering may not be fully realised if predators and parasitoids are not available to control pests damaging developing fruits [6]. Therefore, studies that consider multiple ecosystem services can provide more effective recommendations for biodiversity conservation and agricultural production in agroecosystems than studies focussing on a single service or animal group.

Here, we present one of the first attempts to quantify the net outcome of positive and negative animal activities on crop production, using apples as a case study. Pome fruit production is impacted by a number of vertebrate and arthropod pests [8–10], which directly affect fruit from flowering time (e.g. thrips [11]) through to harvest (e.g. birds [8]). Fruit size and quality are enhanced when insect pollinators are active during flowering [12, 13], and natural enemies [9] and insectivorous birds [14] can enhance yields indirectly by controlling arthropod pests. The net outcome of these activities will determine the final benefit or loss that a grower experiences.

We used a combination of arthropod community sampling and vertebrate exclusion experiments to assess the net outcome of pollination and pest control interactions on final yields under different environmental contexts, *sensu* [4]. We surveyed arthropods across the entire growing season and looked for relationships between measures of yield quantity and quality and arthropod functional groups. Specifically, we addressed the following questions: (i) does vertebrate exclusion from apple branches, from before flowering to harvest, influence yield quantity and quality (as a result of changes in arthropod activity)?; (ii) is the net yield outcome in orchards related to arthropod community composition?; and (iii) how does the net outcome differ across environmental contexts (geographic region and orchard management intensity)?

## Materials and Methods

### Ethics Statement

This study does not require an ethics statement. All sampling was conducted on private land with the permission of owners.

### Study area and system

This study was conducted in three of Australia’s major apple-growing regions: Batlow, New South Wales; Shepparton and Harcourt, Victoria (S1 Fig). All regions differ in elevation, climate and topography (S1 Table). Two orchards were chosen in each region to represent high-intensity and low-intensity management, respectively, resulting in a total of six orchards. All orchards were managed according to organic or integrated pest management (IPM) principles and all cultivated multiple varieties of apples across the orchard. We were unable to study the same apple cultivar in each orchard, due to the logistical challenges of finding suitable orchard pairs in each region, and access restrictions within each orchard. We focused on differences between orchards, rather than within orchards. Therefore, we selected focal trees in each orchard (10 trees per orchard, at least 15 m apart) within a single block of apple trees, to minimise within-orchard environmental effects. All focal blocks were located at the edge of each orchard, near to natural or semi-natural vegetation (unmanaged woodland). Hence, our results are most relevant to interpreting relationships near orchard edges.

### Yield variables

On each focal tree, two branches were randomly chosen as study branches for the duration of the experiment. We paired treatments on a single tree to control for differences between tree biology and orchard microclimates. Pairs of branches were at similar heights and on the same side of the tree (at least 1 m apart). One branch was covered in 15mm diamond mesh vertebrate exclusion netting (hereafter “exclusion”). The other branch remained open for the duration of the experiment (hereafter “open”). Branches were netted to exclude both potential vertebrate predators of arthropods (primarily birds) and possible vertebrate damage agents of apples (primarily birds and mammals). This allowed us to examine the impact of arthropod communities on apple yield in the presence or absence of vertebrate predators and other damage agents, facilitating a more nuanced understanding of the trade-offs in animal activity affecting yield. Branches were netted just prior to flowering, to account for animal activity over the entire fruit development process. The use of exclusion netting is a standard method for studying the effects of plant–arthropod interactions (e.g. pollination, herbivory) in the absence of vertebrate pollinators and predators [2, 15–17]. We did not collect data on flower visitation rates in our study, but *ad hoc* observations of netted branches in each orchard showed that pollinator insects were not deterred from visiting flowers inside nets, and previous studies have also found that vertebrate exclusion nets do not prevent access to plants by arthropods [15–17].

We sampled all sites on three occasions during the growing season: flowering (2–7 October 2014); mid-season, prior to manual thinning (17 November—7 December 2014); and end of season, pre-harvest (19–23 January 2015). The following variables were collected from each study branch: (October) number of blossoms; (November) number of initial fruitlets set; (January) number of final fruit, number of fruit with minor insect damage (1 pinprick hole), and number of fruit with major insect damage (>1 pinprick hole). Initial fruitlet set per branch (“fruit set”) was calculated by dividing the number of fruitlets that developed after flowering ceased, by the number of blossoms open during flowering. Percentage yield loss per branch at harvest (“yield loss”) was calculated as the number of fruit with major insect damage divided by the total number of mature fruit on the branch. For each branch, we also calculated a net yield outcome (“net outcome”) relevant to apple production and saleability by calculating the number of undamaged fruit at harvest as a proportion of the number of blossoms open during flowering (i.e. the number of potential fruit). This value represents the marketable yield (which is determined by the absence of arthropod damage) as an outcome of the net effects of pollination, biological control, pest damage and fruit abortion. Fruit abortion occurs from a variety of abiotic and biotic factors, predominantly resource limitation, weather factors and insect damage [18]. High values (close to 1) indicate relatively high levels of fruit set and fruit development and low levels of pest damage, while low values (close to 0) indicate the reverse. Fruit set, yield loss and net outcome data were non-normally distributed proportions.

### Arthropod sampling

Arthropod communities were sampled at each focal tree on each sampling occasion. All sampling days were fine (sunny or partly cloudy) with maximum temperatures between 17–30°C. Two methods of sampling were used at each site and a single person conducted all rounds of sampling to avoid collector bias. We focused on multiple positive and negative arthropod activities across the growing season, because limiting sampling to one taxonomic group (e.g. bees) can overlook other ecological interactions that also influence yield outcomes [4]. Therefore, sampling was designed to survey a broad cross-section of the arthropod community on and around each tree using standardised methods for collecting arthropods in agroecosystems, rather than to intensively sample a specific taxonomic group.

Pan traps (one yellow, one white) were used to target flying insects (e.g. Diptera and Hymenoptera species), as this is a standard method for collecting these taxa [19]. Because pan traps can attract flying insects from great distances due to contrast against surrounding vegetation [20], we placed traps on the ground at the base of each focal tree to limit attraction to insects flying within the vicinity of that tree. On each sampling occasion, traps were exposed for approximately seven hours during a single day. Insects were stored in ethanol at the Charles Sturt University laboratory. Native bees (Hymenoptera: Apoidea, Anthophila) were identified to species or subgenera using taxonomic keys by Michener [21]. The European honey bee (*Apis mellifera* L.) is an introduced species in Australia and was not included in analyses. We collected very few honey bee individuals (total 7 individuals across all orchards) because our sampling method was designed to target non-*Apis* bees and non-bee pollinators [19, 22]. We were also unable to determine if the collected honey bees were commercially-managed or wild honey bees and, because our main goal was to assess the effects of wild animal activity on apple yield, we removed them from samples; hereafter, ‘bees’ refers to native species only. Recent studies have found that non-*Apis* bees are more effective apple pollinators than European honey bees [23, 24], so it is unlikely that removing the small number of bees from our samples would have affected outcomes of our analyses or our conclusions. Wasps and flies were identified to family and morphospecies [25, 26]. For analyses, we grouped wasp morphospecies into small-bodied ‘parasitoids’ (Hymenoptera: Parasitica and Chrysidoidea families, predominantly parasitoids of insect pest species) that would likely contribute only to biological control, and large-bodied ‘vespids’ (Hymenoptera: Vespoidea families, predominantly predators and parasitoids that also feed on nectar and pollen) that would likely contribute to both pollination and biological control. Fly morphospecies were grouped as hoverflies (Diptera: Syrphidae), calyptrate flies (Diptera: Calyptratae), and other predatory/parasitic flies (Diptera: Stratiomyiidae; Asilidae; Pipunculidae).

Beat samples were also collected from each focal tree by shaking five random branches separately over a 100 x 50 cm rectangular white tray. This is a standard method for collecting non-flying arthropods on vegetation [19]. Branches were shaken gently to avoid damaging flowers and fruit and study branches were not used for any beat sample collection. Arthropods that dropped onto the tray were counted and identified to taxonomic order, or species when recognisable (e.g. apple dimpling bug, *Campylomma liebknechti* Girault). All beat samples were collected in the morning between 0900 and 1200 hours. For both methods, abundances for each arthropod taxon were pooled across the three sampling occasions to create a value of total abundance for each taxon for the growing season.

### Functional groups

Arthropods were sorted into functional groups based on their feeding habits, as this trait is most closely associated with pollination and biological control services [27]. Five main feeding habits were identified: leaf/stem suckers; bud/fruit suckers; pollen/nectar only; insect prey only; pollen/nectar + insect prey (hereafter “pollen+insect”) (S2 Table). Community weighted mean values (CWM) were calculated for each feeding group as:

$$\text{CWM }=\;\sum_{\text{i}=\text{1}}^{\text{S}}{\text{p}}_{\text{i }}{\text{x}}_{\text{i}}$$

where p_i_ is the abundance of species i as a proportion of the total abundance of all species, and x_i_ is the trait value of species i [28]. For each site, we also calculated a ratio of pest:beneficial arthropods (P:B) by dividing the total abundance of pest arthropods (leaf/stem suckers and bud/fruit suckers) by the total abundance of beneficial arthropods (pollen/nectar feeders, insect prey only and pollen+insect). This ratio represents the dominance of each functional group, where values >1 indicate that pest abundance was greater than beneficial abundance, and values < 1 indicate the reverse.

### Data Analysis

#### Relationships between animal activities and yield outcomes

We aimed to identify the net outcome for apple yields across an entire growing season and relate these outcomes to arthropod community composition and landscape context. Net outcome was considered relative to animal activities that directly and indirectly impact fruit development, as well as the environmental context these activities occur in. To account for the nested study design, we used separate generalised linear mixed models (GLMMs) to test for the relationship between each yield parameter (fruit set, yield loss and net outcome) and groups of arthropod community predictor variables. To avoid over parameterisation, three separate models were run for each yield parameter using the following groups of predictors: (i) ‘pest’ arthropod CWMs (leaf/stem; bud/fruit); (ii) ‘beneficial’ arthropod CWMs (pollen/nectar; insect prey; pollen+insect); and (iii) the P:B ratio per tree. In all models, vertebrate pest control treatment (n = 2; open vs. exclusion) was included as a fixed effect and cultivar (n = 7), plus branch (n = 120) nested in tree (n = 60) nested in orchard (n = 6), were included as random effects (S1 File). Variables were rescaled to enable direct comparability of regression coefficients [29, 30].

#### Environmental context

To determine whether net outcomes and arthropod community composition differed across environmental contexts, we examined the local (orchard management intensity) and landscape (geographical regions) effects. Although we had a mix of certified organic and uncertified orchards, management approaches differed greatly between orchards within each context across the study period. Focusing on dichotomous management labels (e.g. organic vs conventional) is not always useful for assessing biodiversity and ecosystem services in agroecosystems, because individual farm management can vary significantly depending on grower’s personal choices and the surrounding landscape [31, 32]. Instead, we ranked orchards along a gradient of ecological management values according to six qualitative and quantitative criteria that distinguish between low-intensity, organic or ecological management vs. high-intensity or conventional management (S3 Table). The six criteria were based on key ecological principles of sustainable farming systems, as outlined in Nicholls & Altieri [33]. Due to the small sample size (six orchards in three regions), we focus on using non-parametric and correlative analyses to identify patterns that may inform future hypotheses, rather than attempting to identify causal relationships.

To identify differences between individual orchards, we focused on the orchard management gradient. We used separate Kruskal-Wallis tests to test for between-orchard differences in the P:B ratio and the median net outcome per branch treatment. We calculated Euclidean distances for net outcomes per branch treatment, and Bray-Curtis distances for P:B ratios, to determine pairwise differences in each variable between orchards. To represent differences in management intensity, we calculated differences between ecological management ratings for each pair of orchards (S3 Table). Spearman correlations were used to identify whether difference in management intensity was correlated with distance between net outcomes. Non-metric multidimensional scaling (NMDS) ordination (based on Euclidean distance) was used to identify the relative associations between sites based on net outcome per branch. To identify if net outcomes (grouped by orchard) matched the gradient of management intensity, minimum spanning trees were applied to each ordination. This is a clustering technique that identifies the shortest path through every point in the ordination space and is thus a measure of connectivity between sites (in this case, based on a gradient of management intensity). To determine if landscape (rather than local) factors were influencing plant-animal interactions, we pooled P:B ratios and net outcomes per treatment by geographical region (Batlow, Harcourt, Shepparton). We used Mann-Whitney pairwise comparisons to identify differences in net outcomes per treatment between pairs of regions. GLMMs were conducted in R (v 3.1.1) [34] and all other analyses were conducted in PAST 3.07 [35].

## Results

### Relationships between animal activities and yield outcomes

**Fig 1:**
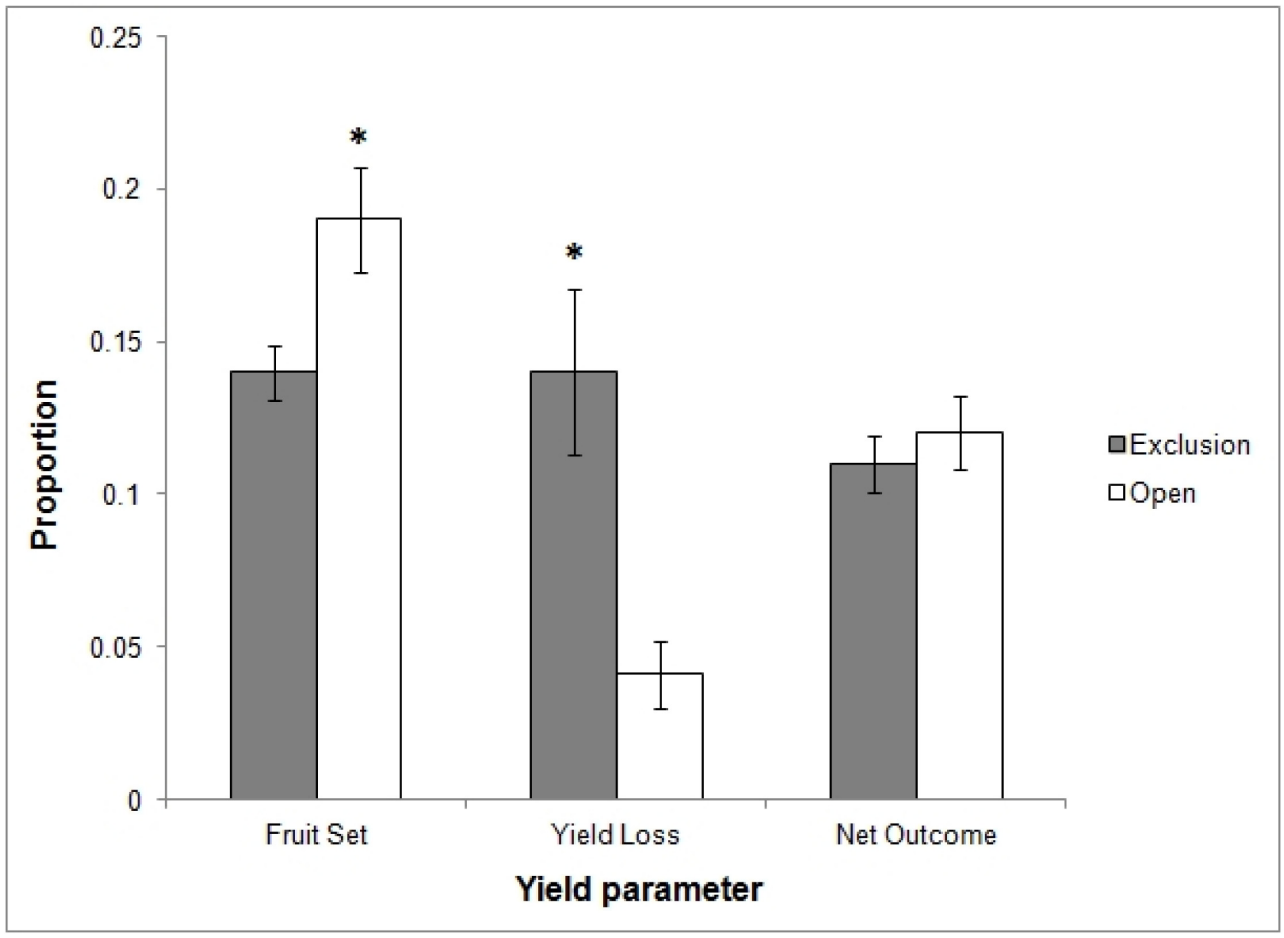
Mean yield parameters (± SE) for open and exclusion branch treatments on each tree (n = 60). Fruit Set = number of initial fruitlets as a proportion of the number of blossoms; Yield Loss = number of damaged fruit at harvest as a proportion of the total number of fruit; Net outcome = the number of undamaged fruit as a proportion of the number of initial blossoms per branch. Asterisks indicate a significant difference between open and exclusion means for the relevant yield parameter (p< 0.05).

Average fruit set was higher on open branches (mean = 19%, SE = 2%) compared to exclusion branches (mean = 14%, SE = 0.1%), while average yield loss (quantified as arthropod damage) was significantly higher on exclusion branches (mean = 14% SE = 3%) compared to open branches (mean = 4% SE = 1%; Table 1, Fig 1). Average net outcome did not differ between open (mean = 12% SE = 1%) and exclusion branches (mean = 11% SE = 1%; Table 1, Fig 1).

**Table 1:** Model parameters for generalised linear mixed models (GLMMs) examining relationships between yield parameters, vertebrate exclusion treatments (Treatment), arthropod feeding groups and the pest:beneficial arthropod ratio (P:B ratio).

Yield parameter	Fixed	Arthropod group	Estimate (SE)	95% CI	R^2^_GLMM(*c*)_	R^2^_GLMM(*m*)_
Fruit Set	Intercept		**-1.93 (0.20)**	-2.39, -1.48		
	Treatment		**0.23 (0.11)**	0.02, 0.44		
		Leaf/stem	-0.12 (0.24)	-0.60, 0.37	0.10	0.005
		Bud/fruit	-0.08 (0.27)	-0.62, 0.45		
		Pollen/nectar	0.11 (0.16)	-0.21, 0.44	0.10	0.01
		Insect prey	-0.23 (0.25)	-0.75, 0.27		
		Pollen+Insects	0.22 (0.21)	-0.20, 0.63		
		P:B Ratio	-0.09 (0.23)	-0.56, 0.38	0.10	0.004
Yield Loss	Intercept		**-4.18 (1.06)**	-6.26, -2.10		
	Treatment		**-2.69 (0.69)**	-4.05, -1.33		
		Leaf/stem	0.77 (1.06)	-1.32, 2.85	0.46	0.10
		Bud/fruit	0.51 (1.22)	-1.89, 2.89		
		Pollen/nectar	**-2.81 (0.90)**	-4.58, -1.04	0.45	0.19
		Insect prey	-0.75 (0.98)	-2.67, 1.18		
		Pollen+Insects	0.77 (0.86)	-0.91, 2.44		
		P:B Ratio	0.62 (1.03)	-1.39, 2.63	0.45	0.09
Net Outcome	Intercept		**-2.28 (0.16)**	-2.65, -1.92		
	Treatment		0.08 (0.11)	-0.15, 0.31		
		Leaf/stem	-0.16 (0.25)	-0.63, 0.36	0.092	0.005
		Bud/fruit	-0.21 (0.26)	-0.72, 0.36		
		Pollen/nectar	0.09 (0.18)	-0.26, 0.45	0.09	0.01
		Insect prey	-0.11 (0.25)	-0.67, 0.36		
		Pollen+Insects	0.37 (0.23)	-0.11, 0.80		
		P:B Ratio	-0.13 (0.26)	-0.62, 0.40	0.09	0.002

Bold text indicates estimates that were statistically significant (P<0.05). SE = standard error for model estimates; 95% CI = 95% confidence intervals; R^2^_GLMM(*c*)_ = amount of variance explained by whole model; R^2^_GLMM(*m*)_ = amount of variance explained by fixed factors.

Conditional R^2^ values showed that our models explained only a small amount of variance in fruit set and net outcome (Table 1), suggesting that other factors not included in the models (e.g. abiotic factors) had a greater effect on these yield parameters. Both fruit set and net outcome were negatively associated with the two pest arthropod groups (leaf/stem & bud/fruit feeders) and predatory arthropods (insect prey), and positively associated with pollen/nectar and pollen+insect feeders; however, none of these relationships were statistically significant (Table 1). In the case of yield loss, approximately 45–50% of variance was explained by the combined effects of all random and fixed effects (e.g. orchard, tree, exclusion treatment and arthropod activity) (Table 1). There was a significant negative relationship between yield loss and pollen/nectar feeders, i.e. higher yield loss was associated with lower community-weighted abundance of native bees (Table 1).

Fruit set and net outcomes per tree had a negative relationship with P:B ratio, i.e. fruit set and marketable yield increased when the abundance of beneficial arthropods was greater than the abundance of pest arthropods (Table 1). Yield loss had a positive relationship with the P:B ratio, i.e. arthropod damage increased when the number of pest arthropods was greater than the number of beneficial arthropods (Table 1).

### Environmental context

**Fig 2:**
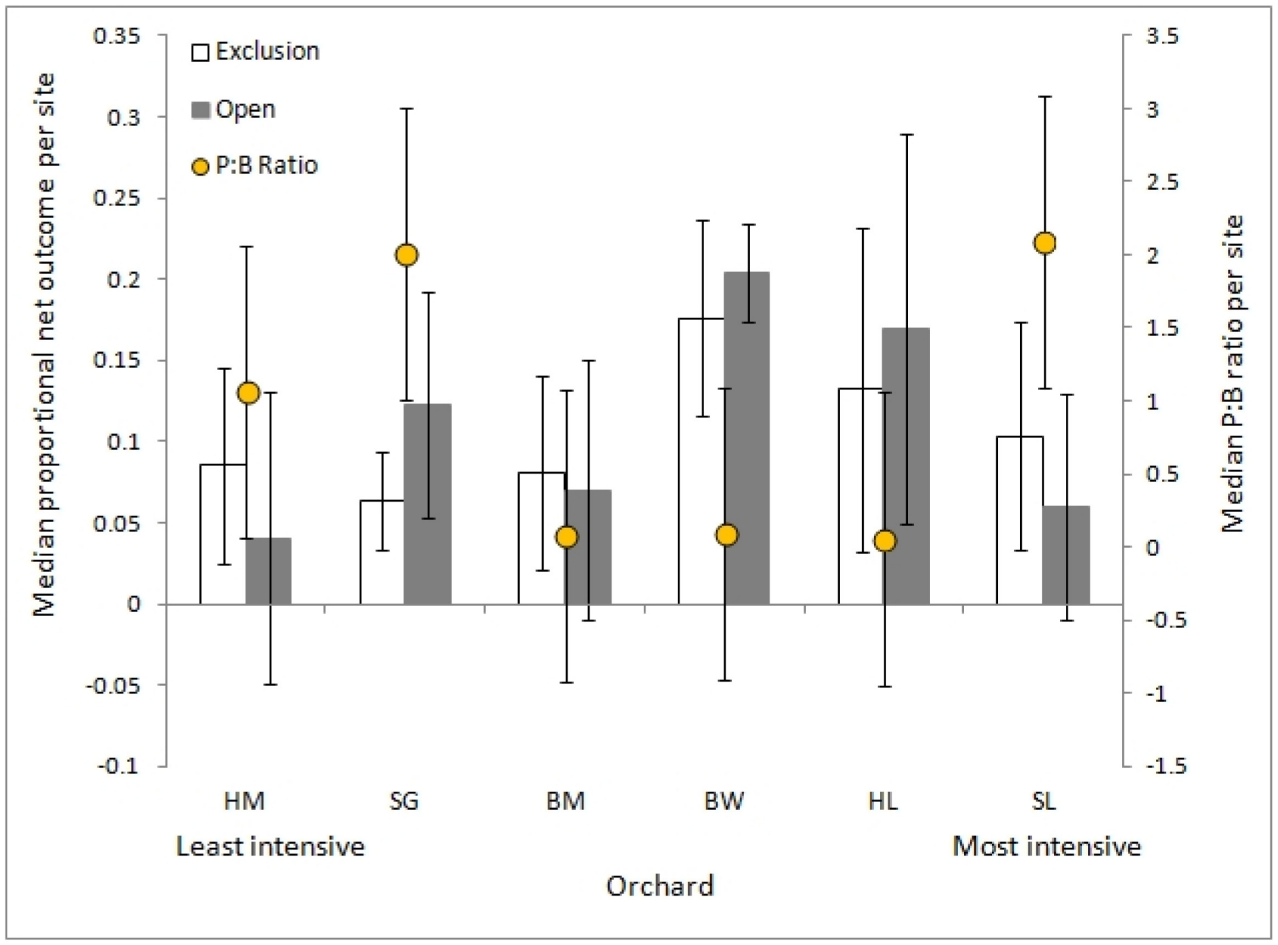
The median (± SD) proportional net outcome of arthropod activity in terms of yield (pollination success—pest damage) and the median (± SD) P:B ratio differed between individual orchards. The net outcome value represents the number of undamaged fruit as a proportion of the number of initial blossoms per branch; the P:B ratio is the ratio of pest arthropods to beneficial arthropods per site, where large values indicate that pest arthropod abundance exceeded beneficial arthropod abundance (S1 and S3 Tables).

**Fig 3:**
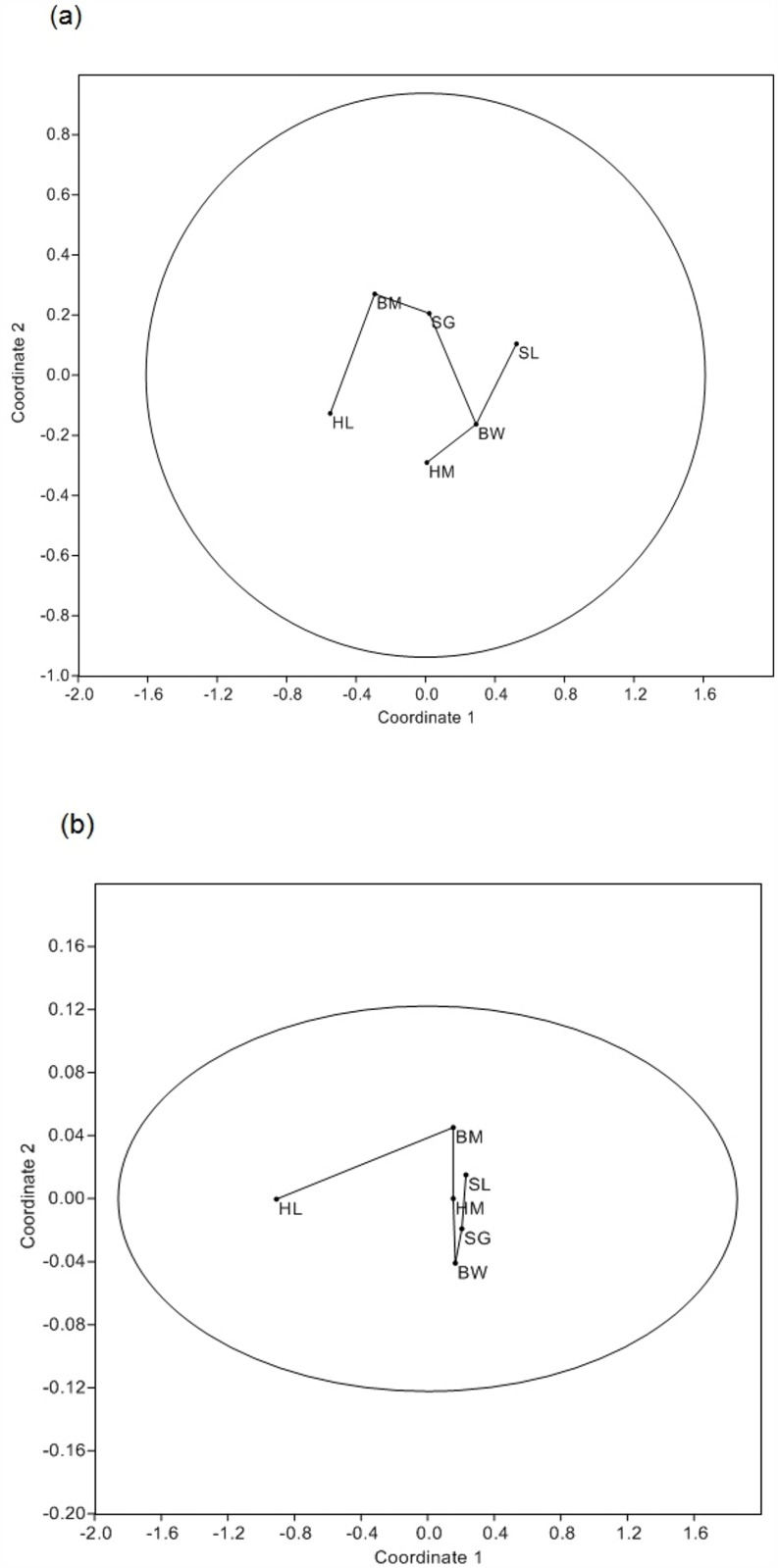
The gradient of net yield outcomes for open (a) and exclusion (b) treatments across orchards did not match the gradient of management intensity. NMDS ordination includes 95% ellipses. N.B. The gradient of orchard management intensity from least to most intensive is: HM, SG, BM, BW, HL, SL (S1 and S3 Tables).

The median net outcome for both branch treatments differed between the six orchards: (open) H = 20.86, p < 0.001; (exclusion) H = 13.29, p = 0.02). The median P:B ratio per tree also differed between orchards (H = 44.08, p < 0.001; Fig 2). For each pair of orchards, neither the pairwise distance between net outcomes, nor the pairwise distance between P:B ratios, was correlated with the pairwise difference in management intensity: (open) r_s_ = 0.09, p = 0.76; (exclusion) r_s_ = 0.01, p = 0.96; (P:B ratio) r_s_ = -0.07, p = 0.82. Ordination showed that net outcomes per branch and P:B ratio for each orchard did not match the gradient of increasing management intensity (Fig 3), i.e. differences in net outcomes and arthropod communities between orchards were not directly associated with differences in management intensity.

There was no difference in the median net outcome per branch between geographical regions: (exclusion) Shepparton, median = 8% SD = 5%; Batlow, median = 11% SD = 6%; Harcourt, median = 13% SD = 9%; H = 5.87, p = 0.05; (open) Shepparton, median = 8% SD = 6%; Batlow, median = 12% SD = 9%; Harcourt, median = 15% SD = 11%; H = 2.13, p = 0.35. The median P:B ratio was significantly different between regions (H = 34.01, p < 0.001), being highest in Shepparton (median = 2.04 SD = 1.02) orchards compared to Batlow (median = 0.10 SD = 0.08; z = -5.40, p < 0.001) and Harcourt (median = 0.29 SD = 0.87; z = -4.07, p < 0.001). There was also a significant difference between the P:B ratio in Batlow and Harcourt (z = -2.21, p = 0.03).

## Discussion

### Relationships between animal activities and yield outcome

We found no difference in the net ecological outcome (in terms of yield) between open and exclusion branch treatments, despite significant differences in fruit set and yield loss. This is a critically important result because it shows that if we had only focussed on one stage of the crop cycle and calculated yield loss as a proportion of mature fruit damaged by arthropod pests (as occurs in many studies of biological control), we would have erroneously concluded that animal (i.e. in this case, vertebrate) activity in apple orchards was providing the ecosystem service of biological control, resulting in increased crop yields for growers. However, by considering the entire growing season, and calculating net fruit yield as the proportion of apple blossoms that resulted in fully-developed, undamaged fruit, we show that the combined impacts of vertebrate and arthropod animal activity had a neutral effect on apple production. That is, the beneficial activities of pollination and biological control were juxtaposed against the detrimental activities of arthropod pest damage.

The lack of difference between paired branch treatments suggests that, across the entire growing season, fruit development on individual apple trees is influenced more by arthropod activity than by vertebrate activity. Arthropod activity directly influences apple fruit development positively and negatively before flowering (e.g. pollen-feeding thrips [36]) during flowering (e.g. insect pollinators [13]), and throughout fruit development (e.g. fruit-damaging pests and their natural enemies [10, 37]).

Complex interactions between arthropod species and other organisms can also influence fruit yield indirectly. Codling moth females may lay more eggs on apples harbouring *Metschnikowia* yeast species [38]; these yeasts are commonly found in nectar, including in apple blossoms [39], and have close associations with some beneficial insects, such as lacewing or bee species [38, 40]. The effects of such complex interactions on fruit yield are yet to be explored in apple orchards, but it is clear that direct and indirect interactions between the activities of multiple arthropods can influence fruit yield across the entire season. In contrast, vertebrates (particularly bird species) occasionally feed on apple fruit, but are less damaging to yields overall than arthropod pest species [36]. Vertebrate activity is likely to have the greatest (positive) effect on production during the fruit development period, through predation on fruit-damaging pests like codling moth [14]. We also found that net outcomes for both treatments differed between individual orchards, suggesting that by focusing only on yield parameters across sites, regardless of orchard context, complex species-environment interactions that also influence yield can be overlooked.

Calculating net outcome per branch showed that studies focusing only on fruit set or fruit damage may not be indicative of the overall effect of animal activity on yield. Exclusion of vertebrates from branches resulted in lower apple fruit set and higher yield loss from arthropod damage. Fruit set was negatively associated with both pest arthropod groups and both pollinator groups (Table 1); however, fruit set models indicated that other factors had more influence on the variance in fruit set. This is not surprising, as fruit set in apple can be influenced by many environmental factors, including weather conditions in previous seasons and the previous year’s fruit set [41]. The pest arthropod species we identified in this study (S2 Table) may not directly influence fruit set. For example, aphid and planthopper species can cause severe damage to apple leaves and shoots, but their effects on fruit set are mostly indirect through declines in overall tree health [36, 42]. Most of the pollinator taxa collected in this study were seen foraging on apple pollen during bloom (e.g. native bees, Syrphidae flies, Calyptrata flies, Tiphiidae wasps), but there is very little information on wild pollinators of apple in Australian agroecosystems. European honey bee colonies are recommended by government agricultural extension services as the best option for optimal pollination in Australian apple orchards [43], but the apple’s flowering strategy and blossom structure mean that other insect pollinators (e.g. smaller insects, or occasional pollen feeders like wasps and flies) may make a more significant contribution to apple pollination [44, 45]. In northern hemisphere apple orchards, some Diptera and non-*Apis* Hymenoptera species can be more effective at pollinating apples than European honey bees [46–49] and there is scope for further detailed research to identify non-honey bee pollinators of apple in Australia. In addition, species in some pollinating fly families (e.g. Syrphidae, Tachinidae) also contribute to pest control, as larval stages are parasites and predators of common crop pests like aphids, thrips and sucking bugs [50, 51].

The higher rates of yield loss on exclusion branches at harvest (quantified as the proportion of fully-developed fruit on each branch that showed major arthropod damage) were most likely a result of reduced biological control of arthropods by insectivorous vertebrates during the fruit development period. Similar effects of vertebrate exclusion on yield quantity and quality have also been found in coffee [2], cacao [52], oil palm [53], and kale [54] production systems. The majority of arthropod damage we found on developed fruit matched the feeding scars known to be caused by bud/fruit feeding arthropods, a group that includes thrips, apple dimpling bug, pest moths (e.g. codling moth, light brown apple moth) and weevils (S2 Table). Some of these taxa can cause damage to mature fruit through their activities on apple blossoms at the flowering stage, rather than a result of direct attack at the mature fruit stage [11]. Predation of thrips by vertebrates is likely to be opportunistic [55] or incidental, for example by nectarivorous birds feeding on thrips-infested flowers [56]. However, insectivorous birds are considered particularly effective at controlling some of the most damaging caterpillar pests of apple fruit, such as codling moth [14, 57, 58]. Hence, our results suggest that vertebrates may be important for controlling arthropod pests during fruit maturation, but may have little effect on arthropod populations during flowering.

The significant negative relationship between yield loss and pollen/nectar feeders (i.e. bees) suggests that arthropod damage to apple fruit increased as the abundance of native bees decreased. Investigation of the raw data found no correlation between bee abundance and pest arthropod abundances (Pearson’s r between -0.18 and 0.08), and it is unlikely that bee abundance would directly influence the activity of fruit-damaging arthropods. Herbivory (including florivory and frugivory) and pollination are known to have combined effects on plant fitness through various pathways, i.e. increased pollination is often related to reduced herbivory, and vice versa [59–61]. However, the mechanics of these interactions are not fully understood and there is scope for further research to investigate how reductions in the availability of pollination services might affect fruit/seed damage. A caveat to interpreting our result is that bee abundance is not a measure of pollination efficiency and our method of calculating yield loss (arthropod-damaged fruit as a proportion of total harvested fruit) did not account for the level of pollination. Therefore, it is most likely that the strong negative relationship between fruit damage and bee abundance reflects broader environmental effects (e.g. orchard management) that may influence ecosystem functions such as fruit development indirectly, by influencing abundances of the animal groups that contribute to those functions.

### Environmental context

Net outcomes per branch and the P:B ratio differed significantly between individual orchards, but neither variable was correlated with the gradient of orchard management intensity. The small number of orchards was not sufficient to identify a causal effect of orchard management, so these results should be interpreted with caution. Sampling more orchards, or including other matrix and management variables in the evaluation, would reveal more about the relationship between net outcomes and management practices. We found that geographic region did not affect net outcome values, but did affect the P:B ratio for each orchard, with the highest ratios found in the Shepparton region orchards (i.e. pest arthropod abundance was much higher than beneficial arthropod abundance in Shepparton). Landscape-scale attributes (e.g. composition, complexity) and regional species pools affect local pest and natural enemy communities and the associated provision of pest control services, but effects can vary across species [62–64], at different levels of landscape simplification [64–66], or as vegetation changes across time [67]. Recent evidence suggests that landscape complexity can also affect pest control in agroecosystems indirectly, by influencing interactions between arthropod and vertebrate natural enemies [68].

Landscape history can also influence composition of plant and animal communities, species responses to current management, and ecosystem services outcomes [69, 70], yet this factor is often neglected in studies of pest control and pollination in agroecosystems. For example, the Shepparton region has a long history of severe resource degradation and native vegetation clearing [71], which has affected bird community composition [72]. This type of landscape-scale intensification can increase pest abundances [73] and reduce the efficacy of biological control [66], but the responses of arthropods to similar factors have not been examined in our study region. Our results are particularly interesting, because both Shepparton orchards have been established for over 100 years in the same locality, but with different management histories. One orchard is certified biodynamic (a specialised form of holistic organic agriculture), yet it had a similar average P:B ratio (> 2) to the other, an intensive, conventionally-managed orchard which ranked the lowest on our ecological management scale (S3 Table). In contrast, the average P:B ratio for orchards across the other two regions was less than 1. Therefore, understanding how broader temporal and spatial contexts influence animal activity on individual farms can be more informative for agroecosystem management than focusing on simple differences between local management factors [74].

## Conclusions

Our study provides a novel approach to research and management of biodiversity and ES in agroecosystems. By calculating net outcomes of multiple ecosystem service-related interactions across the entire growing season, we have shown that measurements of yield quality and quantity based on a limited period of fruit development may not be indicative of the net ecological function of the system. This has important implications for biodiversity conservation and food production in agroecosystems. Harvested fruit yields can be influenced by multiple ecological factors across time [6, 75]. Yet, many studies that aim to quantify the effect of animal activities on yield often overlook the synergistic effect of all these interactions, instead focussing on one period of fruit development, or one positive or negative plant-animal interaction [3]. We encourage researchers and managers to take a holistic approach when measuring animal activity in agroecosystems, and consider how multiple species interact throughout the year, rather than reducing focus to individual plant-animal interactions or fruit development periods. More studies focusing on net outcomes within a social-ecological context, i.e. studies that consider multiple ecological, management and landscape factors, are imperative to advance knowledge of biodiversity-ecosystem services relationships and provide incentive for landscape-scale management of agroecosystems.

## Supporting Information

S1 Fig Map of Australia showing study locations. (PDF)

S1 File Data. (XLSX)

S1 Table Characteristics of study orchards. (PDF)

S2 Table Arthropod taxa included in each feeding trait group. (PDF)

S3 Table Ecological management ranking for study orchards. (PDF)
