# Supplementary figures and images for: Combining Costs and Benefits of Animal Activities to Assess Net Yield Outcomes in Apple Orchards
Source: PLoS One. 2016 Jul 8;11(7):e0158618. doi: 10.1371/journal.pone.0158618 (PMC4938594; doi:10.1371/journal.pone.0158618)

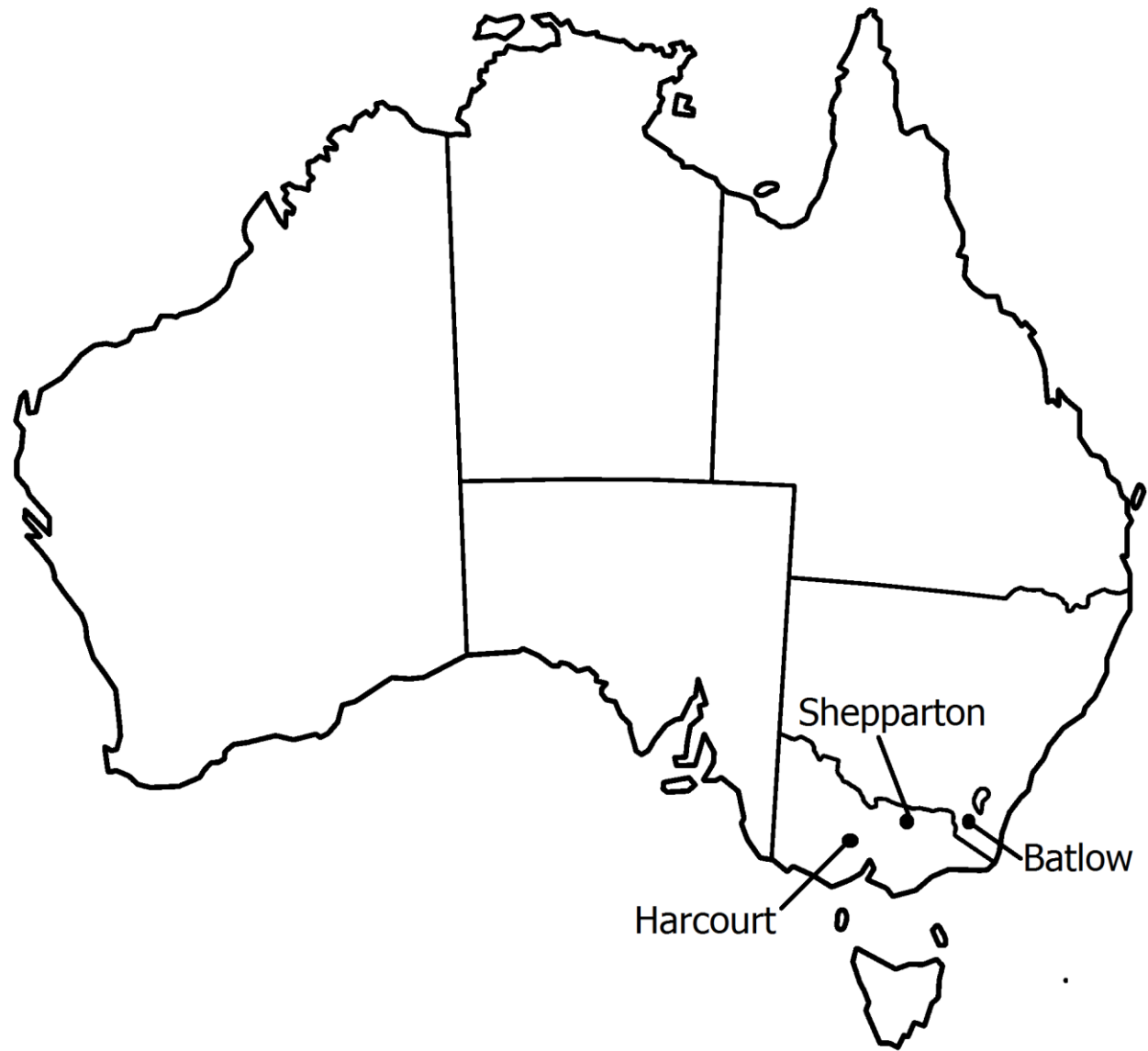

Supplement: S1 Fig — (PDF) [file pone.0158618.s001.pdf]
