# Supplementary material for: Combining Costs and Benefits of Animal Activities to Assess Net Yield Outcomes in Apple Orchards
Source: PLoS One. 2016 Jul 8;11(7):e0158618. doi: 10.1371/journal.pone.0158618 (PMC4938594; doi:10.1371/journal.pone.0158618)
Supplement: S1 Table — (PDF) [file pone.0158618.s003.pdf]

Table S1 Location and characteristics of study orchards

Region & study orchards	Lat, Long	Approx. elevation (m asl)	Climate	Topography	Orchard management	Apple Cultivar
Batlow						
BM	-35.511, 148.099	880	Cool temperate	Rolling hills	IPM	Royal Gala
BW	-35.493, 148.153	775	Cool temperate	Rolling hills	IPM	Royal Gala
Shepparton						
SL	-36,397, 145.295	115	Hot dry summer, cool winter	Floodplain	Conventional	Pink Lady
SG	-36.382, 145.138	110	Hot dry summer, cool winter	Floodplain	Certified biodynamic*	Granny Smith, Golden Delicious
Harcourt						
HM	-37.003, 144.283	417	Cool temperate	Valley, granite outcrops	Certified organic	Cox, Royal Gala, Pink Lady, Granny Smith, Gravenstein
HL	-37.026, 144.277	410	Cool temperate	Valley, granite outcrops	Conventional	Pink Lady, Sundowner, Granny Smith

* Biodynamic farming is a holistic form of organic agriculture that operates on strict organic practices in all aspects of soil, farm and crop management.
