# Supplementary material for: Combining Costs and Benefits of Animal Activities to Assess Net Yield Outcomes in Apple Orchards
Source: PLoS One. 2016 Jul 8;11(7):e0158618. doi: 10.1371/journal.pone.0158618 (PMC4938594; doi:10.1371/journal.pone.0158618)
Supplement: S2 Table — (PDF) [file pone.0158618.s004.pdf]

Table S2 Arthropod taxa in each feeding trait group. Arthropods were grouped by their main effect on apple crop production as a result of their known activity in Australian apple orchards (State of New South Wales 2009).

**Leaf/stem suckers**	**Bud/fruit suckers**	**Pollen/nectar only**	**Insect prey only**	**Pollen/nectar + insect prey**
Planthoppers (Hemiptera: Auchenorrhyncha)	Thrips (Thysanoptera)	Native bees (Hymenoptera: Apoidea)	Parasitoid wasps (Hymenoptera: Parasitica; Chrysidoidea)	Hoverflies (Diptera: Syrphidae)
Aphids (Hemiptera: Aphididae)	Pest moths (Lepidoptera)		Spiders (Aranae)	Vespid wasps (Hymenoptera: Vespoidea)
	True bugs (Hemiptera: Heteroptera)		Lacewings (Neuroptera)	Calyptrate flies (Diptera: Calyptratae)
	Weevils (Coleoptera: Curculionoidea)		Predatory & parasitic flies (Diptera: Stratiomyiidae; Asilidae; Pipunculidae)	
			Predatory beetles (Coleoptera: Coccinellidae)	
			Earwigs (Dermaptera)	

Reference: State of New South Wales (2009) Integrated Pest Management for Australian Apples and Pears. Department of Industry and Investment. Available online: http://www.dpi.nsw.gov.au/agriculture/horticulture/pomes/ipm-apples-pears
