# Supplementary material for: Combining Costs and Benefits of Animal Activities to Assess Net Yield Outcomes in Apple Orchards
Source: PLoS One. 2016 Jul 8;11(7):e0158618. doi: 10.1371/journal.pone.0158618 (PMC4938594; doi:10.1371/journal.pone.0158618)
Supplement: S3 Table — (PDF) [file pone.0158618.s005.pdf]

Table S3 The six study orchards were ranked along a gradient of ecological management (where 1 = most sustainable orchard). A combination of qualitative and quantitative criteria were chosen based on ideal properties of sustainable agroecosystems *sensu* (Nicholls & Altieri 2013). Qualitative criteria were treated as questions with binary responses, where 1 = yes, 0 = no. Quantitative criteria are based on data collected in the field (M. E. Saunders, unpubl. data). Scores were summed for each orchard to achieve an ecological management rating, where high values indicate sustainable, ecological management. Orchards were then ranked from 1-6 (least intensive—most intensive) based on these scores.

**Ecological Management Factor**	**HM**	**SG**	**BM**	**BW**	**HL**	**SL**
Certified organic?	1	1	0	0	0	0
Weedy ground cover?	1	1	1	1	1	1
Ground cover floral diversity/m^2	1.1	1.7	1.5	1.03	1.13	0.4
Ground cover (%)/m^2	1	0.92	0.91	0.75	0.59	0.89
Unmanaged woodland within 100m?[+]	1	1	1	1	1	0
Small blocks of apple trees <100m^2?	1	0	1	1	1	0
Ecological Management Rating	6.1	5.62	5.41	4.78	4.72	2.29
Gradient ranking	1	2	3	4	5	6

[+] This is the common native vegetation structural type for study regions. Some woodland patches had a mix of native and exotic tree and shrub species, but all were unmanaged and structurally similar.
